# The CD14 rs2569190 TT Genotype Is Associated with an Improved 30-Day Survival in Patients with Sepsis: A Prospective Observational Cohort Study

**DOI:** 10.1371/journal.pone.0127761

**Published:** 2015-05-28

**Authors:** Ashham Mansur, Benjamin Liese, Maximilian Steinau, Michael Ghadimi, Ingo Bergmann, Mladen Tzvetkov, Aron Frederik Popov, Tim Beissbarth, Martin Bauer, José Hinz

**Affiliations:** 1 Department of Anesthesiology, University Medical Center, Georg August University, Goettingen, Germany; 2 Department of General and Visceral Surgery, University Medical Center, Georg August University, Goettingen, Germany; 3 Institute of Clinical Pharmacology, University Medical Center, Georg August University, Goettingen, Germany; 4 Department of Cardiothoracic Transplantation & Mechanical Support, Royal Brompton and Harefield Hospital, Harefield, London, United Kingdom; 5 Department of Medical Statistics, University Medical Center, Georg August University, Goettingen, Germany; University of Florida College of Medicine, UNITED STATES

## Abstract

According to previous investigations, CD14 is suggested to play a pivotal role in initiating and perpetuating the pro-inflammatory response during sepsis. A functional polymorphism within the CD14 gene, rs2569190, has been shown to impact the pro-inflammatory response upon stimulation with lipopolysaccharide, a central mediator of inflammation in sepsis. In this study, we hypothesized that the strong pro-inflammatory response induced by the TT genotype of CD14 rs2569190 may have a beneficial effect on survival (30-day) in patients with sepsis. A total of 417 adult patients with sepsis (and of western European descent) were enrolled into this observational study. Blood samples were collected for rs2569190 genotyping. Patients were followed over the course of their stay in the ICU, and the 30-day mortality risk was recorded as the primary outcome parameter. Sepsis-related organ failure assessment (SOFA) scores were quantified at sepsis onset and throughout the observational period to monitor organ failure as a secondary variable. Moreover, organ support-free days were evaluated as a secondary outcome parameter. TT-homozygous patients were compared to C-allele carriers. Kaplan-Meier survival analysis revealed a higher 30-day mortality risk among C-allele carriers compared with T homozygotes (p = 0.0261). To exclude the effect of potential confounders (age, gender, BMI and type of infection) and covariates that varied at baseline with a p-value < 0.2 (e.g., comorbidities), we performed multivariate Cox regression analysis to examine the survival time. The CD14 rs2569190 C allele remained a significant covariate for the 30-day mortality risk in the multivariate analysis (hazard ratio, 2.11; 95% CI, 1.08-4.12; p = 0.0282). The 30-day mortality rate among C allele carriers was 23%, whereas the T homozygotes had a mortality rate of 13%. Additionally, an analysis of organ-specific SOFA scores revealed a significantly higher SOFA-Central nervous system score among patients carrying the C allele compared with T-homozygous patients (1.9±1.1 and 1.6±1.0, respectively; p = 0.0311). In conclusion, CD14 rs2569190 may act as a prognostic variable for the short-term outcome (30-day survival) in patients with sepsis.

## Introduction

Worldwide, sepsis is one of the most frequent complications due to infection among critically ill patients and is increasing in prevalence [1–3]. Although according to the Surviving Sepsis Campaign, mortality from sepsis has decreased due to improved supportive care and evidence-based guidelines for diagnosis and timely intervention [4, 5], mortality remains at approximately 30% [4], with higher mortality rates in developing countries [6, 7]. Lipopolysaccharide (LPS) or endotoxin, the major component of the outer membrane of gram-negative bacteria, plays a major role in initiating the pro-inflammatory response associated with sepsis [8]. LPS triggers inflammation in gram-negative sepsis, as well as in gram-positive and fungal sepsis, when excessive amounts of gut-derived LPS are released during intestinal hypoperfusion [9, 10]. LPS binds membrane or soluble CD14 (sCD14) and the myeloid differentiation-2 (MD-2)-TLR4 complex, resulting in NF-kB activation and the production of IL-6, IL-1b, TNF and type I interferons [11–13]. Although CD14 was initially described as the essential co-receptor mediating the LPS activation of monocytes, subsequent investigations have shown that it also participates in immune cell activation by gram-positive cell-wall components, such as peptidoglycan [14]. Findings of numerous investigations suggest a pivotal role for CD14 in initiating and perpetuating the pro-inflammatory response during the course of sepsis [14–16]. The pro-inflammatory response is essential to eradicate primary infections and prevent the acquisition of secondary infections in patients with sepsis [17].

A functional polymorphism located at position -159 in the promoter region of the CD14 gene rs2569190 has been shown to influence the pro-inflammatory response upon stimulation in human leucocytes [18], is associated with transcriptional activity of the promoter and can also affect the production of sCD14 and tumor necrosis factor α [19]. Furthermore, the TT genotype of rs2569190 has been associated with a stronger pro-inflammatory response and higher CD14 transcriptional activity [18, 19]. Several studies have previously investigated the association between CD14 rs2569190 and the susceptibility to sepsis, as well as the outcome of sepsis, and found controversial results [16]. Given that CD14 may play an essential role in the pro-inflammatory response, we hypothesized that the strong pro-inflammatory response induced by the TT genotype of CD14 rs2569190 may improve survival (30-day) in patients with sepsis.

The aim of the study was achieved; in accordance with our hypothesis, patients with the TT genotype showed an improved 30-day survival compared with C-allele carriers.

## Materials and Methods

### Patients

Between April 2012 and June 2014, adult patients of European descent admitted to the intensive care units (ICUs) at the University Medical Center Goettingen (UMG) were screened daily for sepsis, severe sepsis and septic shock according to standard criteria [20]. European descent was determined by questioning the patients, their next of kin or their legal representatives. The patient exclusion criteria have been described previously elsewhere [21, 22] and comprised pre-existing diseases, immunosuppressive status and medications that may modulate the inflammatory response in patients with sepsis. The study was approved by the University of Goettingen ethics committee, Goettingen, Germany (15/1/12) and conformed to the Declaration of Helsinki ethical principles (Seoul, 2008). Written informed consent was obtained either from patients or their legal representatives.

### Data collection

Mortality risk within 30 days of sepsis onset was recorded as the primary outcome parameter. Secondary outcome variables included organ dysfunction, which was evaluated using the Sequential Organ Failure Assessment (SOFA) score during the 28-day observational period in the ICU [23]. Ventilator-free days, vasopressor-free days and dialysis-free days were also recorded as secondary variables. Patients were further followed up for a maximum of 90 days, and the 90-day mortality was recorded as a secondary outcome variable. The length of ICU stay, several laboratory parameters and microbiological findings were also recorded as secondary endpoints. Relevant clinical data were collected from the electronic patient record system (IntelliSpace Critical Care and Anesthesia (ICCA); Philips Healthcare, Andover, Massachusetts, USA); all relevant medical records, including microbiological findings and medications, are available in this system. Preexisting conditions and medical histories were determined by examining the physicians’ notes; questioning the patients, their next of kin or their legal representatives; and by consulting the patient’s family doctor.

### CD14 rs2569190 genotyping

DNA was extracted by automated solid phase extraction from 350 μl EDTA whole blood using an EZ1 DNA Blood Kit in BioRobot EZ1 or from PBMCs using an AllPrep DNA Mini Kit according to the manufacturer’s instructions (all from Qiagen, Hilden, Germany). The DNA quantity and quality were determined spectrophotometrically. Genotyping was performed using the pre-designed TaqMan SNP genotyping assay C__16043997_10 according to the manufacturer’s instructions (Life Technology, Darmstadt, Germany).

A total of 15% of the samples were genotyped in duplicate, yielding results that showed complete concordance. The observed genotypes were in Hardy-Weinberg equilibrium. The identity of the DNA samples was controlled by sex-typing and showed 100% concordance between the initially documented and the genetically determined sex [24].

### Statistical analyses

The Hardy-Weinberg equilibrium was performed using a chi-square test. Statistical analyses were performed with Statistica (version 10, StatSoft, Tulsa, Oklahoma, USA). The significance was based on contingency tables and calculated using Fisher’s exact or chi-square tests, as appropriate. Two continuous variables were compared using the Mann-Whitney test. Time-to-event data were compared using Cox's F-test from the Statistica function survival. P-values less than 0.05 were considered significant. To exclude the effects of potential confounders (age, gender, BMI and type of infection: gram-negative, gram-positive and fungal) and covariates that varied at baseline with a p-value < 0.2 (e.g., comorbidities), we performed a multivariate Cox regression analysis to examine the survival time.

## Results

### Patients

A total of 417 adult patients of western European descent with sepsis were enrolled in this prospective investigation. Of these patients, 279 were male (67%), and 138 were female (33%) (Table 1). The patient median age was 65. The genotype distribution of CD14 rs2569190 was 117:225:75 (CC:CT:TT), which is consistent with Hardy–Weinberg equilibrium (p = 0.1833). The minor allele frequency was 45%. The CD14 rs2569190 CC and CT genotypes were pooled together to explore the clinical impact of the TT genotype compared with that of C-allele carriers in accordance with our *a priori* hypothesis (Table 1). The sepsis subtypes included sepsis/severe sepsis (39%) and septic shock (61%). The morbidity scores, SOFA and APACHE II, at baseline were 9.2±4.0 and 21.5±7.3, respectively (Table 1). The comorbidities included hypertension, a history of myocardial infarction, chronic obstructive pulmonary disease (COPD), renal dysfunction, diabetes mellitus, chronic liver diseases, a history of cancer, and a history of stroke (Table 1). Moreover, recent surgical history, site of infection and organ support (mechanical ventilation, vasopressor therapy and renal-replacement therapy) were also recorded at baseline (Table 1).

**Table 1:** Patient baseline characteristics according to CD14 rs2569190 genotype.

	All(n = 417)	CT/CC(n = 342)	TT(n = 75)	P value
Age [years]	63±15	63±15	64±15	0.4123
Male, %	67	67	68	0.8241
Body-mass index	28±7	28±7	28±8	0.9366
Severity of sepsis				
Sepsis/severe sepsis, %	39	39	43	0.5169
Septic shock, %	61	61	57	0.5169
SOFA score	9.2±4.0	9.3±4.0	8.7±4.2	0.3340
APACHE II score	21.5±7.3	21.6±7.3	21.0±7.3	0.6236
Comorbidities, %				
Hypertension	58	59	53	0.3877
History of myocardial infarction	7	6	11	0.1987
COPD	17	16	23	0.1324
Renal dysfunction	12	11	12	0.8834
Diabetes mellitus (NIDDM)	10	10	8	0.5562
Diabetes mellitus (IDDM)	12	11	16	0.2712
Chronic liver diseases	7	7	7	0.9138
History of cancer	19	18	23	0.3312
History of stroke	6	6	7	0.7867
Recent surgical history, %				0.3618
Elective surgery	30	28	36	
Emergency surgery	51	52	48	
No history of surgery	19	20	16	
Site of infection, %				0.4953
Lung	54	55	48	
Abdomen	27	26	29	
Bone or soft tissue	5	5	5	
Surgical wound	2	1	4	
Urogenital	2	2	4	
Primary bacteremia	7	7	4	
Other	4	4	5	
Organ support, %				
Mechanical ventilation	84	84	84	0.9860
Use of vasopressor	61	61	57	0.5134
Renal-replacement therapy	10	9	11	0.7271

The data are presented as the means ± SD or percentages

### Disease severity at baseline

No differences in age, gender, or body mass index were found between the two groups of study subjects. Furthermore, no differences were found in the SOFA and APACHE II scores at the onset of sepsis according to the CD14 rs2569190 genotype. Additionally, the comorbidities were equally distributed between TT patients and C-allele carriers. Moreover, there was no difference with respect to organ support (mechanical ventilation, vasopressor therapy and renal-replacement therapy) between the two groups (Table 1).

### Mortality analysis

**Fig 1:**
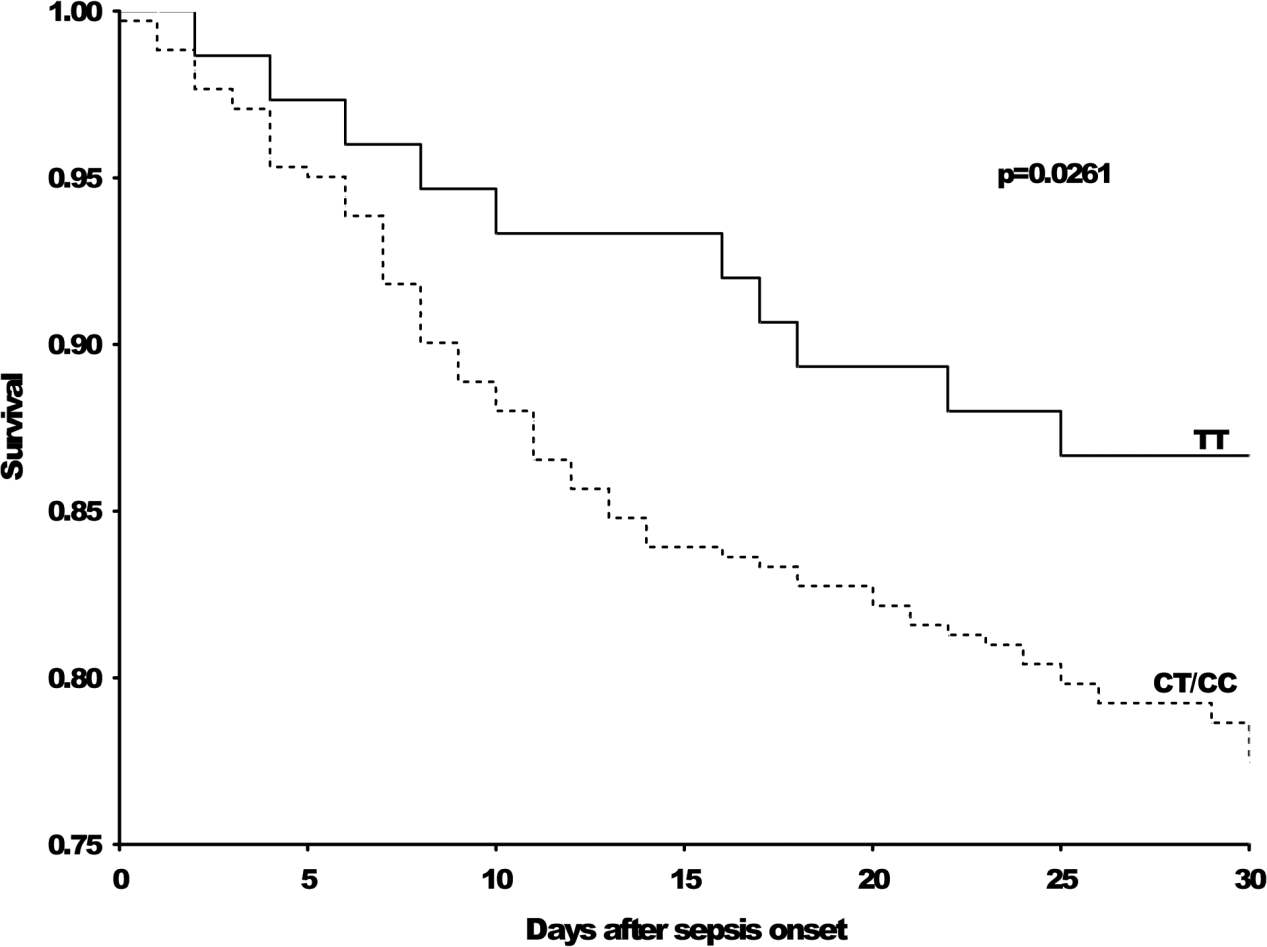
Kaplan-Meier survival analysis. The Kaplan-Meier curve shows the survival curves censored at day 30 for the CD14 rs2569190 TT and CT/CC genotypes. Within this patient sample, the mortality risk was higher among CT/CC patients than among patients with a TT genotype (p = 0.0261, Cox's F-test).

Kaplan-Meier survival analysis revealed a higher 30-day mortality risk among C-allele carriers compared with T-homozygous patients (p = 0.0261) (Fig 1). Within the observational period of 30 days after sepsis onset, 77% of the C-allele carriers had survived, whereas the T homozygotes had a survival rate of 87% (Table 2). An analysis of 90-day mortality revealed no significant difference between the two groups (p = 0.2297) (Table 2).

**Table 2:** Disease severity over the course of sepsis.

	All(n = 417)	CT/CC(n = 342)	TT(n = 75)	P value
SOFA score	6.8±3.6	7.0±3.7	6.0±3.1	0.0918
SOFA-Respiratory score	1.9±0.8	1.9±0.8	1.8±0.8	0.2215
SOFA-Cardiovascular score	1.5±1.0	1.5±1.0	1.3±0.8	0.2468
SOFA-Central nervous system score	1.9±1.1	1.9±1.1	1.6±1.0	0.0311
SOFA-Renal score	0.8±1.2	0.9±1.2	0.7±1.2	0.5202
SOFA-Coagulation score	0.3±0.6	0.4±0.6	0.3±0.5	0.5071
SOFA-Hepatic score	0.4±0.7	0.4±0.7	0.3±0.6	0.5180
Mortality analysis, %				
Death at day 30	21	23	13	0.0490
Death at day 90	31	32	27	0.2297
Length of ICU stay (days)	17±15	17±15	18±17	0.6259
Organ support-free days:				
Vasopressor-free (days)	11±7	10±7	11±7	0.1734
Ventilator-free (days)	5±5	5±5	6±6	0.1547
Dialysis-free (days)	14±8	14±8	14±8	0.7254
ECMO-free (days)	15±9	15±9	15±8	0.5240
Inflammatory values				
Leucocytes (1000/μl)	14±5	14±5	14±5	0.9162
CRP (mg/l) (n)	151±84 (208)	148±85 (169)	165±81 (39)	0.2097
Procalcitonin (ng/dl) (n)	4.5±10.7 (361)	4.1±10.3 (293)	6.0±12.6 (68)	0.0654
Laboratory values				
Lactate (mmol/l)	1.7±1.1	1.7±1.2	1.6±0.6	0.2092
Bilirubin (mg/dl)	1.3±2.2	1.3±2.3	0.9±0.9	0.1844
GOT (IU/l)	209±725	238±804	90±116	0.1961
GPT (IU/l)	106±224	113±243	73±87	0.2341
Kidney values				
Creatinine (mg/dl)	1.3±1.0	1.3±1.0	1.2±0.8	0.8254
Creatinine clearance	103±68	102±65	108±81	0.8614

The data are presented as the means ± SD or percentages.

### Multivariate analysis

To exclude the effects of potential confounders (age, gender, BMI and type of infection: gram-negative, gram-positive and fungal) and covariates that varied at baseline with a p-value < 0.2 (e.g., comorbidities), we performed a multivariate Cox regression analysis to examine the survival time. The CD14 rs2569190 C allele remained a significant covariate for 30-day mortality risk in the multivariate analysis (hazard ratio, 2.11; 95% CI, 1.08–4.12; p = 0.0282) (Table 3). This finding indicates that, despite baseline differences in certain variables (i.e., history of myocardial infarction and COPD) and in other potential confounders (age, gender, BMI and type of infection: gram-negative, gram-positive and fungal), the CD14 rs2569190 C allele remained a prognostic variable with a significant effect on the short-term outcome (30-day survival) in our cohort of sepsis patients (Table 3).

**Table 3:** Cox regression analysis.

Variable	Hazard ratio	95% CI	P value
Age	1.03	1.01–1.05	P<0.0001
Gender, Male	1.63	0.99–2.67	0.0515
BMI	1.02	0.98–1.05	0.2283
History of myocardial infarction	0.94	0.43–2.08	0.8959
COPD	1.17	0.68–1.98	0.5596
Gram-negative infection	0.68	0.43–1.06	0.0919
Gram-positive infection	0.74	0.44–1.24	0.2612
Fungal infection	0.74	0.48–1.15	0.1920
CD14 rs2569190 C allele	2.11	1.08–4.12	0.0282

### Disease severity

During their stay in the ICU, C-allele carriers presented higher mean SOFA scores than did T homozygotes (7.0±3.7 and 6.0±3.1, respectively; p = 0.0918) (Table 2). An analysis of organ-specific SOFA scores revealed a significantly higher SOFA-Central nervous system score among patients carrying the C allele than among T-homozygous patients (1.9±±1.1 and 1.6±1.0, respectively; p = 0.0311) (Table 2). The remaining organ-specific SOFA scores did not differ between the two groups. In addition, an analysis of the organ support-free days (ventilator-free, vasopressor-free and dialysis-free days) showed no differences between C-allele carriers and T homozygotes (Table 2). Moreover, the distribution of infection type (gram-negative, gram-positive, fungal and viral) did not vary between the two studied groups (Table 4). Infection type was determined based upon confirmed culture results.

**Table 4:** Infection types over the observational period.

	CT/CC(n = 342)	TT(n = 75)	P value
Infection type			
Gram-negative	68%	59%	0.1393
Gram-positive	81%	76%	0.3450
Fungal	56%	53%	0.7979
Viral	11%	7%	0.3000

### Inflammatory values

An analysis of inflammatory values revealed higher c reactive protein (CRP) levels among the TT patients compared with the C-allele carriers (165±81 and 148±85, respectively; p = 0.2097). Furthermore, procalcitonin levels were higher among the TT homozygotes compared with the C-allele carriers (6.0±12.6 and 4.1±10.3, respectively; p = 0.0654).

## Discussion

This investigation addresses the hypothesis that sepsis patients with the TT genotype of CD14 rs2569190 may have an improved 30-day survival compared with patients carrying the C allele. Our observation of a significantly improved 30-day survival among CD14 rs2569190 TT patients confirms our hypothesis. The 30-day mortality rate among C allele carriers was 23%, whereas the T homozygotes had a mortality rate of 13%. To the best of our knowledge, these findings underscore for the first time the beneficial potential clinical impact of the CD14 rs2569190 TT genotype on 30-day survival in a cohort of septic patients of exclusively western European descent while remaining consistent with observations from Brazilian cohorts that also revealed a beneficial effect of the TT genotype on the survival of critically ill patients [25–27]. Similarly, our findings are in accordance with studies that showed an increased risk of death among CD14 rs2569190 CC critically ill patients with burn injury [28, 29].

A particular strength of this observation lies in the fact that, despite baseline differences in certain variables (i.e., history of myocardial infarction and COPD) and in other potential confounders (age, gender, BMI and type of infection: gram-negative, gram-positive and fungal), the CD14 rs2569190 C allele remained a prognostic variable with a significant effect on the short-term outcome (30-day survival) in our cohort of sepsis patients. After including the morbidity scores APACHE II and SOFA into the multivariate Cox-regression analysis, the CD14 rs2569190 C allele remained a significant covariate for 30-day mortality risk (hazard ratio, 2.16; 95% CI, 1.09–4.26; p = 0.0265). The resulted hazard ratio (hazard ratio, 2.16) for the C allele is marginally higher than the respective hazard ratio without APACHE II and SOFA scores (hazard ratio, 2.11; 95% CI, 1.08–4.12; p = 0.0282).

The biological correlation of this beneficial clinical association of the TT genotype with the 30-day survival could be attributed to previous functional observations demonstrating a significantly stronger pro-inflammatory response among TT patients compared with C-allele carriers [18, 19]. Although statistical significance was not reached, this argument is supported by the observation of a trend of higher inflammatory values (CRP and procalcitonin) among TT patients compared with C-allele carriers during the ICU stay. This assumption is in conformance with the increasingly prevailing opinion among researchers that the major problem faced by sepsis patients is a predominant state of immunosuppression characterized by a reduced pro-inflammatory status and increased anti-inflammatory response [17]. However, our results are not consistent with previous investigations demonstrating a beneficial effect of the CC genotype on septic shock survival and other studies that found no association between the CD14 rs2569190 genotype and survival among patients with sepsis [15, 16]. An explanation for this inconsistency may be that most of the previous studies had a sample size that was too small to detect the impact of the TT genotype on the survival of patients with sepsis.

The lack of a significant association between the CD14 rs2569190 TT genotype and 90-day mortality suggest little or no biological impact of the TT genotype on long-term mortality. As shown by our group, the 90-day mortality risk among patients with sepsis is related to the functional programmed cell death 1 genetic polymorphism rs11568821 GG genotype [30], which has been shown to influence transcriptional activity [31] and increase PD-1 expression [32]; PD-1 is thought to play an important role in the so-called sepsis-induced immunosuppression [17].

A major advantage of our study was that we report the first investigations of organ-specific dysfunction over the clinical course of disease with regard to CD14 rs2569190 genotypes using organ-specific SOFA scores. The significantly higher SOFA-Central nervous system scores among C-allele patients indicate a pronounced neurological or consciousness impairment in this group. This effect might be attributed to the fact that C-allele carriers, who have been shown to express less LPS-neutralizing sCD14 [33], may have compromised LPS-clearance compared with T homozygotes, making the C-allele carriers more susceptible to neurological dysfunction due to higher serum LPS levels. This explanation is in line with reports in animal models showing that the peripheral injection of LPS induces alterations in neurobehavioral performance and cognitive dysfunction [34, 35]. However, this finding has to be interpreted carefully because the SOFA-Central nervous system score can be affected by the sedative medications given to critically ill patients, which is a potential confounding factor.

One possible limitation to this study is that it focused on the clinical impact of CD14 rs2569190 exclusively; therefore, we cannot exclude that other functional SNPs located in nearby genes and in linkage disequilibrium with the CD14 rs2569190 SNP are responsible for the observed phenotypic effects on the 30-day mortality risk. A further potential limitation of this study is measurement bias, e.g., several clinical parameters (e.g., blood pressure, heart rate, and respiratory rate) are automatically recorded in the electronic patient record system, and we cannot exclude measurement errors with absolute certainty. However, all clinical records were repeatedly checked for plausibility before beginning the statistical analysis.

In conclusion, this is the first study to show a beneficial association of the CD14 rs2569190 TT genotype with short-term survival (30-day) in a cohort of sepsis patients of exclusively western European descent. According to our observations, it is worthwhile to consider CD14 rs2569190 genetic variants in future studies that investigate the genetic risk stratification for short-term mortality in patients with sepsis. Further validation in other ethnic groups is recommended.
